# Ecological risk evaluation and sensitivity analysis of heavy metals on soil organisms under human activities in the Tibet Plateau, China

**DOI:** 10.1371/journal.pone.0285116

**Published:** 2023-08-03

**Authors:** Xia Zeng, Cai Deng, Ying Liang, Juanlin Fu, Shaoxuan Zhang, Tianhua Ni

**Affiliations:** 1 School of Geography and Ocean Science of Nanjing University, Nanjing, China; 2 School of Geography and Resources Science, Neijiang Normal University, Neijiang, China; 3 School of the Environment, Nanjing University, Nanjing, China; 4 Southwest University of Science and Technology, Mianyang, China; Alagappa University, VIET NAM

## Abstract

The Tibetan Plateau (TP), once considered a pristine environment, is now facing increased heavy metal pollution due to human activities, causing unprecedented ecological risks to soil organisms. However, little is known about the sensitivity and tolerance of different soil organisms to heavy metal toxicity in the high-altitude areas of the TP under the background of human activity intensity and future risk control priorities. In this study, we conducted an ecological risk assessment and threshold calculation for 10 heavy metals in soil for typical soil organisms, including Cd, Co, Cr, Cu, Ni, Pb, Zn, Mn, Sb, and Sn, using the species sensitivity distribution (SSD) method in the zone between Ranwu town and Renlongba glacier on the TP. The results revealed that most sampling sites had average levels of Cd, Sb and Ni exceeding their regional soil environmental background values and were the major contaminants. Impressively, the hitherto undeveloped Renlungba Glacier showed relatively high contamination levels of Sb and Ni. From the view of sensitivity differences, the toxicity risks of Cd, Cr, Cu, Ni and Pb were higher for terrestrial plants than for soil invertebrates based on the fitted heavy metal SSD curve trends. In terms of the ecological risk level, the average potentially affected fraction values of Zn and Ni reached 18.5% and 17.0%, respectively, with significant ecological risk at a few sampling sites. In terms of ecological risk thresholds, the Cd concentration at the 5% hazard concentration (HC_5_) control level was 0.05 mg/kg, which was the heavy metal with the highest risk in this study. Comparing the HC_5_ values of each heavy metal with the limit values in the current Chinese soil environmental quality standards, the existing administrative policies as a whole lack a powerful prevention of the potential ecological risk posed by heavy metals to soil organisms.

## Introduction

In recent years, soil heavy metal pollution has become one of the global environmental problems faced by many countries, including China, due to a combination of natural factors and anthropogenic activities of escalating intensity [1, 2]. A national scale survey in 2014 showed that the exceedance rate of soil pollution in China reached 16.1%, of which inorganic pollution, such as heavy metals, was the main cause, with the number of exceedance points accounting for 82.8% [3]. The pollution of heavy metals in the soil is attracting major concern across the China.

As heavy metals are not easily detectable, irreversibly damaged and highly biotoxic, terrestrial plants and animals, as important components of soil ecosystems, are the first to suffer from the threat of soil heavy metal toxicity [4, 5]. Heavy metals produce a variety of inhibitory effects on plants, such as root toxicity, plant dwarfism, decreased yield, and reduced fruit quality, and even endanger human health through the food chain [6, 7]. Animals in the soil, especially invertebrates, are equally difficult to spare. Due to the large number of species, small activity range, and sensitivity to heavy metals, soil animals are significantly affected by the toxicity of heavy metals in terms of individual growth and development and population size [8]. An increasing number of methods for soil heavy metal pollution analysis, including the pollution index method, the enrichment index method, the Nemerow index method, neural networks, geo-accumulation index, etc., have been employed in different regions [9, 10]. Statistical-, pollution index-, and Nemerow index-based methods were exploited to study the heavy metal pollution of China’s Raoyanghe Wetland by Wang, the results shown that deep layer soils were classified as having a ‘mild’ level of pollution [11]. Xiao used geo-accumulation index to evaluate heavy metal pollution for agricultural soil and found that Cd and Hg were the main heavy metal pollutants in agricultural soil of the Guangzhou-Foshan urban zone, South China [12]. Wang analyzed the fractions of Cd in soil along three altitudinal transects on Gongga Mountain and found that Cd reached a moderate contamination level on the eastern and southern transects, but no or slight contamination on the western transect, and the Cd had a low non-carcinogenic risk and no carcinogenic risk despite of adults or children [13]. Nevertheless, studies have mainly focused on the evaluation of soil heavy metal pollution, distribution characteristics and human health risk assessment, but not enough attention has been given to the ecological risk of heavy metals to soil organisms, which are important components of soil ecosystems and the most likely direct receptors of toxicity. While there is a lack of knowledge about the sensitivity of terrestrial organisms to heavy metal toxicity, the ecological risk threshold variation, the proportion of potentially affected species and their relationship with human activities, resulting in a lack of scientific objectives and effective measures for realistic soil heavy metal ecological risk management.

The species susceptibility distribution (SSD) constructs dose-effect relationships between contaminants and species from a toxicological perspective and is an effective method for quantitative ecological risk assessment toward toxic receptors [14]. SSD method is the most recognized method for taking full account of biodiversity and ecosystem integrity, carrying out qualitative and quantitative analysis of risk, and having accurate evaluation results [15, 16]. On the one hand, it was widely used to quantitatively assess the risk probability of heavy metals’ toxic effects on organisms. Xu analyzed the probabilistic ecological risk assessment of moderately polluted areas in Changsha-Zhuzhou-Xiangtan, Hunan Province, by SSD model and the joint probability curve method, and demonstrated that Cd in soil had a certain risk effect on the ecosystem. On the other hand, SSD model was extensively applied to establish ecological thresholds for heavy metals in soils. Liu investigated the short-term toxic effects of Cr(VI) and Cr(III) on the root growth of eleven terrestrial plants, the corresponding fifth percentile hazardous concentrations (HC_5_) by the best fitting SSD curves based on the tenth percentile effect concentrations (EC10) were determined to be 0.60 and 4.51 mg/kg for Cr (VI) and Cr (III), respectively, which provided useful toxicity data for deriving national or local soil quality criteria for trivalent and hexavalent Cr.

Furthermore, few studies focused on the heavy metal of soil in cold, high-altitude, remote, ecologically sensitive and fragile areas. Indeed, heavy metal pollution in soil covers not only towns, industrial and mining lands and arable lands where the population is mainly concentrated, but even the remote and sparsely populated Tibetan Plateau (TP) is not spared [17]. The TP is known as the "third pole of the world" and the "water tower of Asia" due to its extremely high altitude and harsh climatic conditions and is the most important ecological barrier in Southeast Asia. However, the ecological environment of the region is very fragile, which is aggravated by increasingly severe soil heavy metal pollution [18]. Existing studies have shown that in addition to background soil values(The process of rock weathering into soil is also a redistribution process of heavy metal migration and enrichment, which affects the background value of soil heavy metal content) [19], long-range transport by atmospheric circulation [20] and increasingly active anthropogenic activities such as mining, transportation and tourism are important drivers of the increasingly severe heavy metal pollution in the TP. It has been shown in the literature that soils in extensive areas of the TP, such as the central, southeastern and northeastern parts, have been contaminated with heavy metals to some extent, with a few areas suffering from heavier levels of contamination. Cd, Sb and Zn are the main heavy metal contaminants [17]. Therefore, it is necessary to understand the distribution characteristics of heavy metals in TP, and their contamination level, ecological risk threshold variation, the variability of toxic responses among different groups of soil organisms to their toxicity.

The study was conducted for typical terrestrial plants and invertebrates, and the area from Ranwu Town to Renlongba Glacier in the TP was selected as the study area. SSD was applied to carry out dose-effect relationship fitting between soil organisms and heavy metals, quantitative evaluation of soil heavy metal pollution and ecological risk, and calculation of risk level under different impact probabilities. The sensitivity and tolerance of different soil organisms to heavy metal toxicity in the high-altitude areas of the TP under the background of human activity intensity and future risk control priorities are discussed in detail.

## Materials and methods

### Study area and sample collection

**Fig 1:**
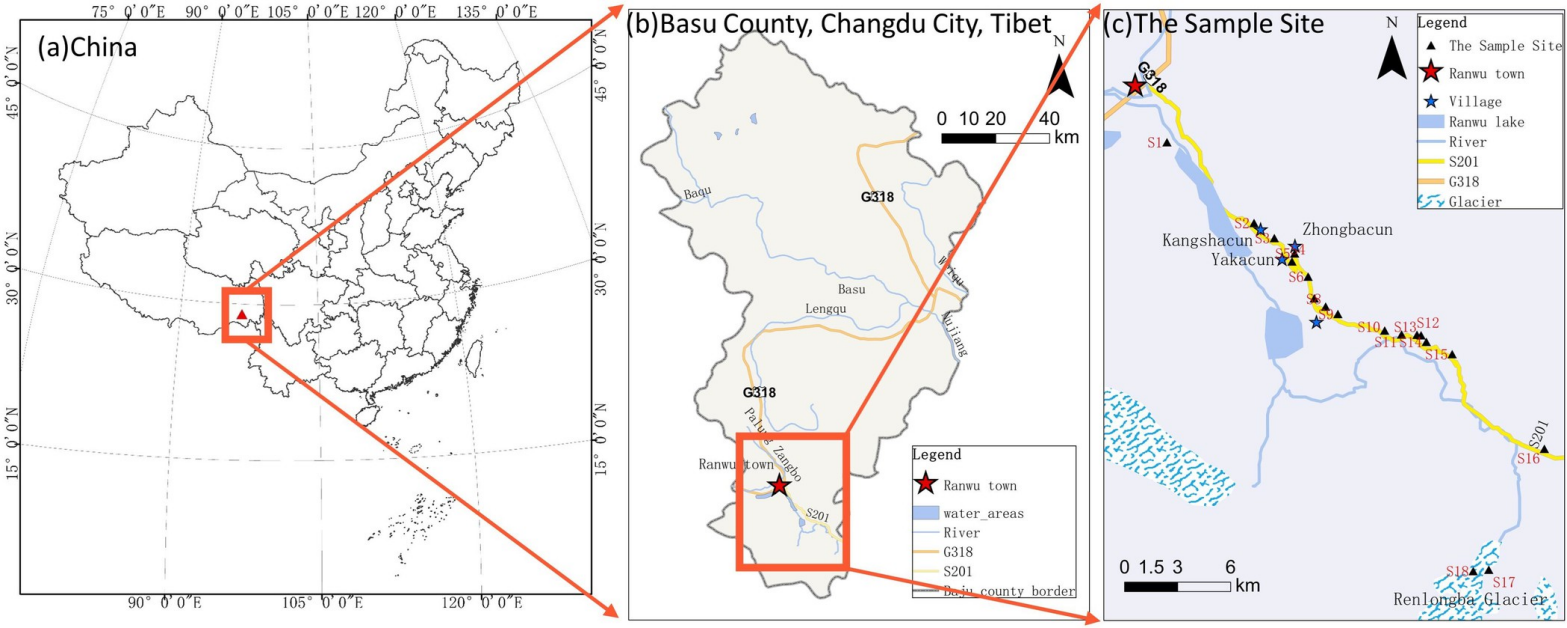
Location and sample sites of the study area.

The study area (96°77′-96°93′E, 29°25′-29°43′N) is shown in Fig 1 and is located along the Rangwu town to Renlongba glacier in the TP, with an altitude span of 1000 m (from 3900 m to 4900 m). The study area is relatively rich in mineral resources, with a wide distribution of volcanic and metamorphic rocks, and is one of the most important mineralized belts in China. The town of Ranwu Lake at the southern edge of the region is a famous tourist attraction due to its famous "western paradise" and is relatively densely populated, with active agricultural production and mining as well as tourism. To the south is the Renlungba Glacier, which is less disturbed by humans. Therefore, the intensity of human activities in the study area from south to north is gradually reduced from strong to weak. The study area is ecologically fragile and sensitive due to its special geography, geology and environmental climate and has been polluted and damaged to a considerable extent [21]. Eighteen sampling sites were set up, labeled S1, S2, S3……, in May of 2021. In each sampling area, soil samples were collected from three sampling points using the S-shaped distribution method, and then the upper and lower soil samples from the three sampling points were mixed thoroughly and stored in a plastic bag. Finally, soil samples from each sampling area were obtained using the quadrat method. The sampling area belongs to the regular observation area of the Southeast TP Comprehensive Observation and Research Station of the Chinese Academy of Sciences and sampling did not require any permissions or approvals of any authorities and did not involve endangered or protected species.

### Laboratory analysis of samples

The sample was evenly spread on a white porcelain plate with a thickness of 2-3cm, placed in a well-ventilated room without direct sunlight, and dried at room temperature of 22 degrees Celsius for approximately 48 hours. The grinding process was performed using a white ceramic mortar, with a sieve size of 2 mesh and 100 mesh. After the initial grinding, the sample was sifted through a 2 mesh sieve, and the sifted portion was mixed before continuing with a 100 mesh sieve. The milled soil sample (0.1 g) with 2 mL concentrated HNO_3_ and 1 mL concentrated HClO_4_ was digested with a microwave digestion device (GEMs). The residue was dissolved in 2 mL of 4 mol/L HCl and diluted to 10.00 ± 0.01 g [22]. The samples were heated on a hot plate at 200°C and kept at a slight boiling state until the solid residue disappeared. Concentrations of ten heavy metals were measured by ICP–OES (Perkin Elmer Optima 5300DV, USA) and by ICP–MS (X-7, Thermoelemental, USA).

Quality assurance and quality control (QA/QC) procedures were implemented by the analysis of a standard reference material obtained from Center for National Standard Reference Material of China. Reagent blanks were monitored throughout the analysis and were used to correct the analytical results [23]. Only Sn was used as an internal standard in the determination of all metal concentrations, and the internal standard element was Rh. A recovery study was performed for each metal to more precisely evaluate the accuracy and reliability of their concentrations [24]. The results are presented in Table 1.

**Table 1:** Measured concentrations and recoveries for heavy metals.

Metal	Certified value (mg/ kg)	Uncertainty	Detected value (mg/ kg)	Recovery (%)
Cd	0.065	0.012	0.058	89
Co	11.6	0.3	11.4	98
Cr	57	3	59	104
Cu	18.3	0.8	17.8	97
Ni	26	1	27	104
Pb	26	2	25	96
Zn	59	2	61	103
Mn	755	13	763	101
Sb	0.5	0.05	0.48	96
Sn	2.6	0.2	2.4	92

### Analysis methods

#### Enrichment factor method

The enrichment factor (EF) is commonly used to distinguish the natural and anthropogenic sources of elements deposited in certain regions and to evaluate the degree of pollution caused by anthropogenic emissions [18]. EF is defined as the concentration ratio of a specific element to the crust element in the sample to the concentration ratio of the corresponding element in the crust [25]. In this work, the concentrations of Mn and other heavy metals (Cd, Co, Cr, Cu, Ni, Pb, Zn, Sb and Sn) in Tibetan soils were used as a reference element and the background value, respectively. According to the factor EF, the degree of contamination was divided as shown in Table 2 [26].

**Table 2:** Pollution judgment standard of enrichment factor.

Grade	Enrichment factor(EF)	Pollution degree
**Ⅰ**	EF<2	Pollution-free
**Ⅱ**	2≤EF<5	Moderate pollution
**Ⅲ**	5≤EF<20	Significant pollution
**Ⅳ**	20≤EF<40	High pollution
**Ⅴ**	EF>40	Extreme pollution

#### Species sensitivity distribution

SSD was applied to evaluate the ecological risk of soil heavy metals in the study area. The ssd tools package of R3.5.2 was used to establish the species sensitivity distribution model [27]. The toxicological data used in this paper were partly obtained from the databases of relevant domestic and foreign institutions, e.g., the ECOTOX toxicity database of the US EPA, OECD eChemPorta, etc. Considering the amount of toxicity data required to construct SSD curves, the chronic toxicity (the chronic maximum none effect concentration (NOEC) and the minimum effective concentration (LOEC)) data of six heavy metals, including Cd, Cr, Cu, Ni, Pb and Zn, were selected to construct SSD curves based on the data collection results in this paper.

SSD curves for different heavy metals on soil organisms were fitted by using a six-function fit model, including burrIII3, gamma, log-gumbel (lgumbel), log-logistic (llogis), log-normal (lnorm) and Weibull, and the best model that was used to calculate HC_5_ was the one with the lowest Akaike’s information criterion corrected (AICC) [28]. To reduce the number of cases due to small data sets or due to data sets that do not satisfy the parameter estimation and to depict the uncertainty in model fitting, nonparametric bootstrap estimation was used to obtain a 95% confidence interval and calculate thresholds for the ecological risk of heavy metals. Based on this, a nonparametric method was selected to fit SSD curves for toxicological data of heavy metals on terrestrial plants and soil invertebrates.

#### Classification of ecological risk levels

The potentially affected fraction (PAF) based on environmental concentrations of pollutants is used to characterize the ecological risk of an ecosystem, with reference to the observations made by Yan et al. in the Liaohe River basin of China [29]. We set up a four-level ecological risk classification system according to PAF, including Level I (at potential risk, assuming more than 5% of species may be threatened by heavy metal toxicity), Level II (at slight risk, assuming more than 15% of species may be threatened by heavy metal toxicity), Level III (at significant risk, assuming more than 30% of species may be threatened by heavy metal toxicity), and Level IV (at serious risk, assuming more than 50% of species may be threatened by heavy metal toxicity).

## Results and discussion

### Variations in heavy metal concentrations

**Fig 2:**
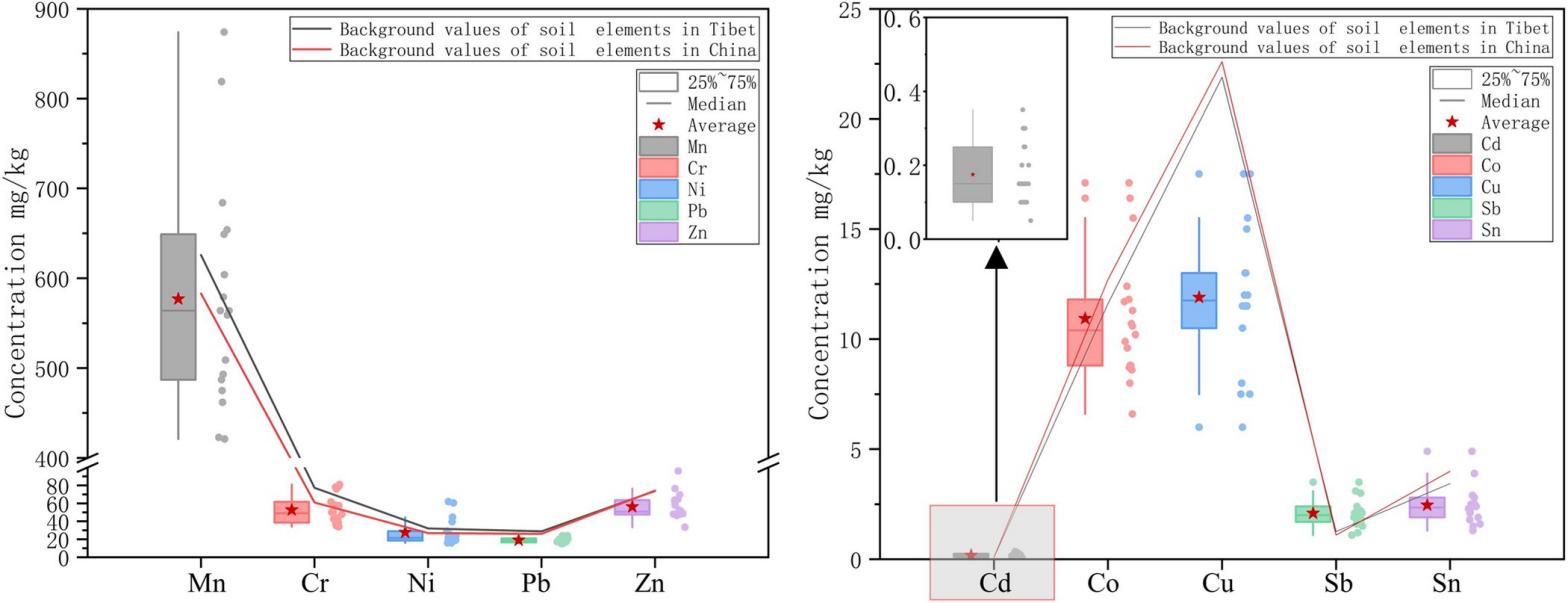
Variation characteristics of heavy metal concentration.

Changes in the concentrations of 10 heavy metals at sampling sites in the study area are shown in Fig 2. Additionally, the background concentrations in Tibetan soils and in Chinese soils are presented. Depending on the mean contents of 10 elements, the order from highest to lowest was Mn, Zn, Cr, Ni, Pb, Cu, Co, Sn, Sb and Cd. In general, there was a very wide range of variation in concentration. In addition to Pb and Cu, whose concentrations from 18 sample sites were lower than the background values, the remaining heavy metal concentrations were higher than the background at some sampling points and lower than the background at other sampling points, while the average contents of Cd, Sb and Ni were higher than the background content, especially Cd, whose maximum concentration was higher than the background values of Cd in Tibet by more than four times, which was essentially the same as the previous findings by Bing et al. [30] and Hou et al. [31]. It can be found that the differences in the spatial distribution characteristics of heavy metal concentrations in the study area are complex, on the one hand influenced by the soil-forming parent rocks [32], which are the main cause of the differences in the background values of heavy metals in different areas. On the other hand, rock weathering or erosion will release heavy metals into the environment, thus affecting the concentration of heavy metals in the environment. However, the differences in measured heavy metal concentrations were much higher than the differences between the background values, as can be seen that heavy metal contamination was complexes in the natural environment due to non-natural factors [24].

**Fig 3:**
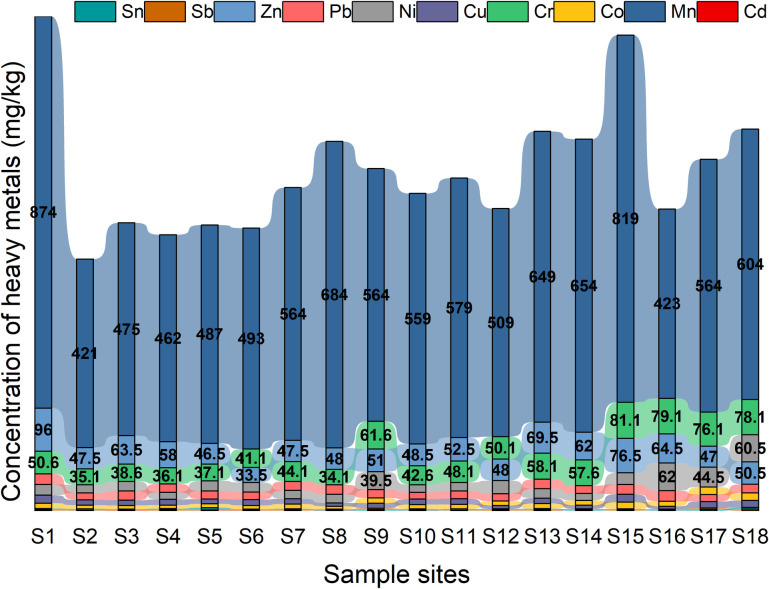
Spatial characteristics of heavy metal concentration.

Fig 3 illustrates the variation in heavy metal concentrations based on the spatial distribution of sampling sites. Overall, the concentrations of Mn, Cu, Co, Cr, Ni, and Zn varied widely, with high and low concentrations varying greatly among different sampling points. In contrast, the changes in Pb, Sn, Sb, and Cd exhibited small variations. In terms of the spatial inhomogeneities in concentration, in one scenario, the highest concentrations of Mn, Pb and Zn were observed in S1, which was close to Ranwu town. In another scenario, we found that the maximum concentrations of Co, Sb and Sn were presented in S18, which was located in Renlongba Glacier, while the highest concentrations of the remaining heavy metals, including Cr, Ni, Cd and Cu, were found at the sampling point along Provincial Road S201. Although the intensity of human activities gradually decreased in the spatial direction from Ranwu Lake town to Renlungba Glacier, cases of soil heavy metal concentrations exceeding background values occurred at different altitudes. Cd was the main exceeder at low altitudes, most likely due to, for example, transportation and agricultural production. Sb and Ni were the exceeders at higher elevations, probably due to interregional atmospheric transport and glacial ablation.

Comparing the heavy metal concentrations of area studied with different regions of the world in the literature (Table 3) revealed that the spatial distribution of soil heavy metals had obvious regional characteristics on a global scale, which was controlled by several factors that can influence the content of heavy metals in soils. First of all, the geology factors, for instance, types of rocks, are the key factors to influent the natural occurrence of heavy metals in soil. The second is anthropogenic activities, such as mining, industrial processes, and the use of fertilizers and pesticides that can introduce heavy metals into the soil, even cause contamination of the soil with heavy metals. Then, soil properties imply that he physical and chemical properties of the soil can influence the concentration of heavy metals in the soil. In addition, climate and biological factors can also affect the content of heavy metals in soils. Overall, the content of heavy metals in soil is influenced by a complex interplay of natural and anthropogenic factors. However, a common feature is that the concentrations of heavy metals in the soil exceed their respective background values to some extent, including in Asia, Europe, and Africa. Ultimately, it can be draw that it is important to note that the concentration of heavy metals in soil can have significant implications for organisms health and the environment, so regular monitoring and management of soil quality is essential.

**Table 3:** Mean concentrations of heavy metal(mg/kg) from different regions of the world and this study.

Heavy metal	This study	Urban soil of Poznan (Poland) [33]	Turnasuyu in Urdu (Turke) [24]	City of Lisbon, (Portugal) [34]	the soils volcanic island in the Indian Ocean [35]	Natural Soils of Jeju Island, (Korea) [36]	Soils of Yelagiri Hills, Tamilnadu, India [37]	Topsoils (Bangkok) [38]
**Cd**	0.18	0.2	0.27	0.42	0.15	0.24	-	-
**Co**	10.9	15.7	6.74	-	-	36	13.9	-
**Cr**	52.74	56.3	5.67	51.5	165.9	73.4	48.1	26
**Cu**	11.89	19.8	19.1	-	52.9	20.1	-	42
**Ni**	27.56	19.1	4.35	62.4	92.1	52.3	32.61	25
**Pb**	18.97	27.7	60.23	8.49	-	14.4	-	48
**Zn**	56.14	57.1	33.06	-	146.1	33.3	71.2	118
**Mn**	576.89	552	361	-	-	729.5	553	-
**Sb**	2.07	0.47	-	-	-	8.99	-	-
**Sn**	2.44	1.1	-	-	-	1.15	-	-

*- no data

### Assessment of heavy metal contamination

#### Extent of heavy metal contamination

**Fig 4:**
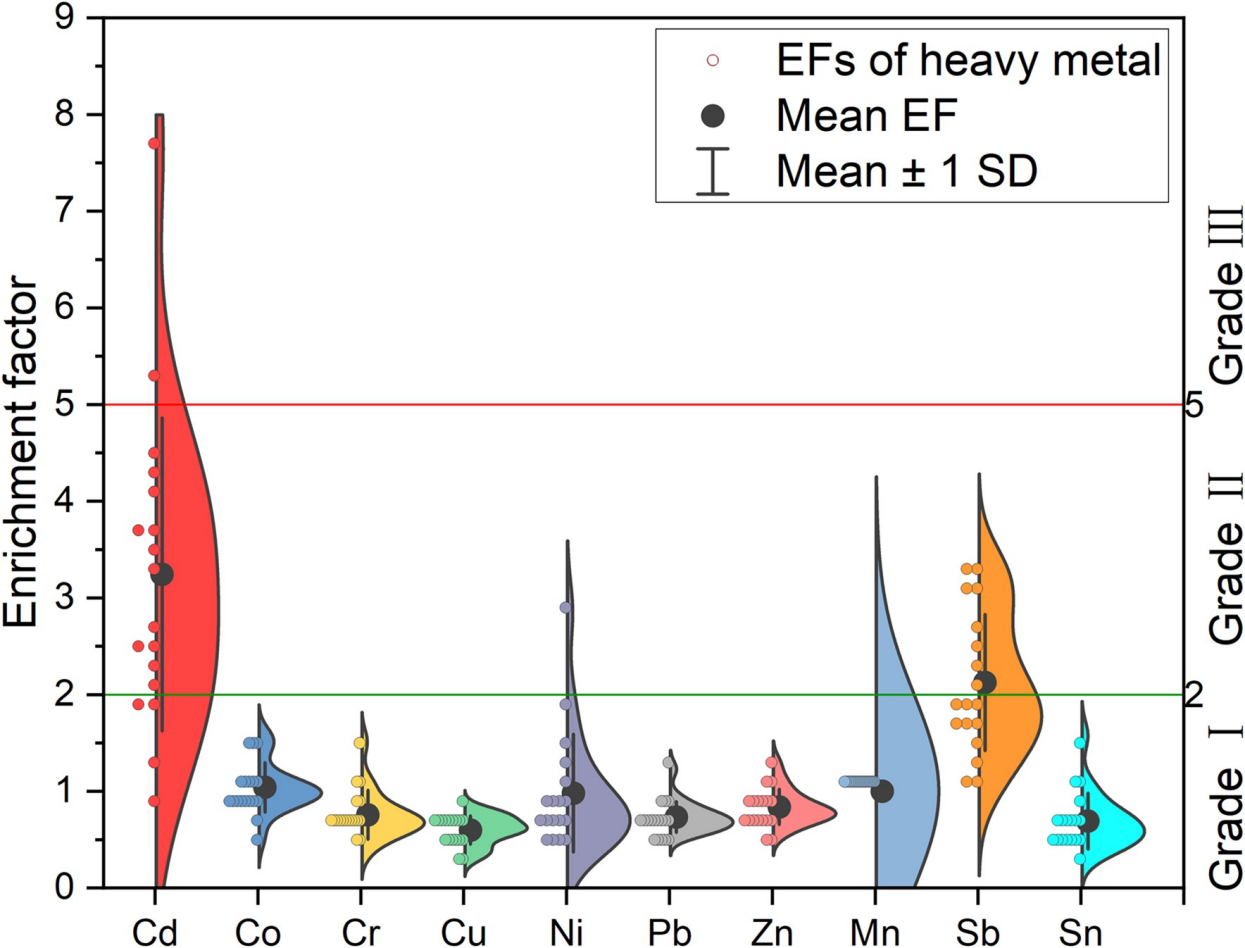
Variation characteristics of heavy metal enrichment factor.

The EFs of heavy metals were shown in Fig 4. The general trend can be observed that for most of the heavy metals except for Cd, Sb and Ni, EFs from 18 sample sites were less than 2, which are in the pollution-free level of grade I. Remarkably, the maximum EF value of Cd was observed to be as high as 7.69, which reached the significant pollution level, and the maximum EF values for both Sb and Ni exceeded 2, which were 3.30 and 2.86, respectively, reaching a moderate pollution degree. Wang utilized the ground accumulation index method to evaluate the degree of heavy metal contamination in surface soils on the TP, and the results showed that it was mainly affected by Cd, Sb and Ni [39]. In the case of average EFs, Cd and Sb were between 2 and 5, reaching a moderate level of contamination, and the remaining heavy metals were less than 2. Therefore, it could be speculated that soil in the study area was dominated by Cd, Sb and Ni contamination, and some spots with peak concentrations may be significantly polluted. Yang et al. used the enrichment factor, geoaccumulation index, and Nemerow synthesis index to evaluate the surface soil heavy metal contamination in the TP and found that Cd and Sb contamination dominated the TP topsoil [40], which was consistent with the trend observed in this study.

#### Spatial inhomogeneities of pollution degree

**Fig 5:**
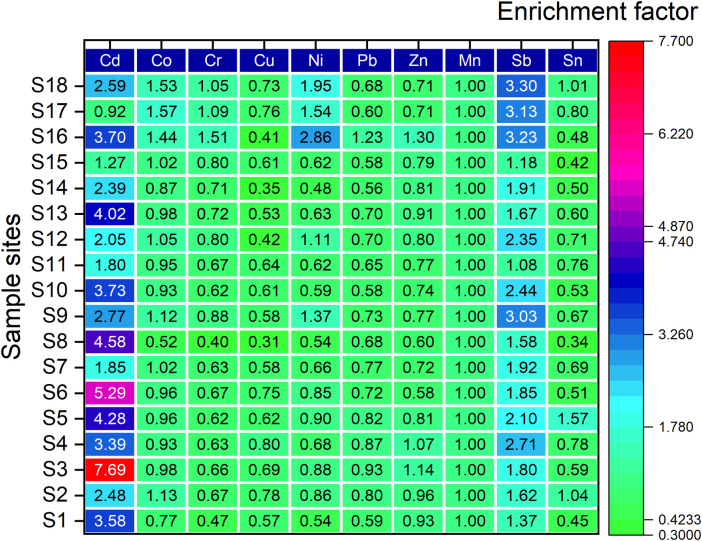
EF heatmaps of heavy metals.

EFs of ten heavy metal for each sample site are presented in Fig 5. Collectively, the EFs of the heavy metals showed less spatial fluctuation, while more pronounced fluctuations were observed for Zn, Ni and Cu. Among them, the EFs of Cd ranging from 0.92 to 7.69 presented a significant spatial fluctuation. The EFs of Ni and Sb ranged from 0.48 to 2.86 and from 1.08 to 3.30, respectively. In the spatial direction from Ranwu town to Renlungba glacier, in addition to natural geographic factors, such as soil type, temperature, wind conditions, etc., the differences are relatively obvious. The intensity of human activities, including resident population, agricultural production, transportation and tourism, is gradually decreasing and is likely to be an important external cause of the changes affecting the heavy metal content.

From the contamination characteristics of different sampling sites, the majority of sampling sites close to the highway had significant Cd and Sb contamination, which reached the level of Class II and Class Ⅲ classified in this study. This trend is similar to the results of the study by Lin et al. [41] on the evaluation of soil heavy metal contamination on both sides of the highway. Compared with other sampling sites, sites numbered S17 and S18, located at the hitherto undeveloped Renlongba Glacier, had relatively high levels of Sb and Ni contamination. On the one hand, it is possible that heavy metal contaminants from human activities in other regions may affect the glacial environment by deposition after long-distance atmospheric transport [42]. On the other hand, snow, as a carrier of information on climate and environmental changes, has some capacity to capture heavy metals in the atmosphere [43], which may contaminate soils in glacial areas, for example, through precipitation or release during glacial melting due to climate change [21].

### Ecological risk assessment of heavy metals in soil

#### Variability of HC_5_ values for heavy metals

**Fig 6:**
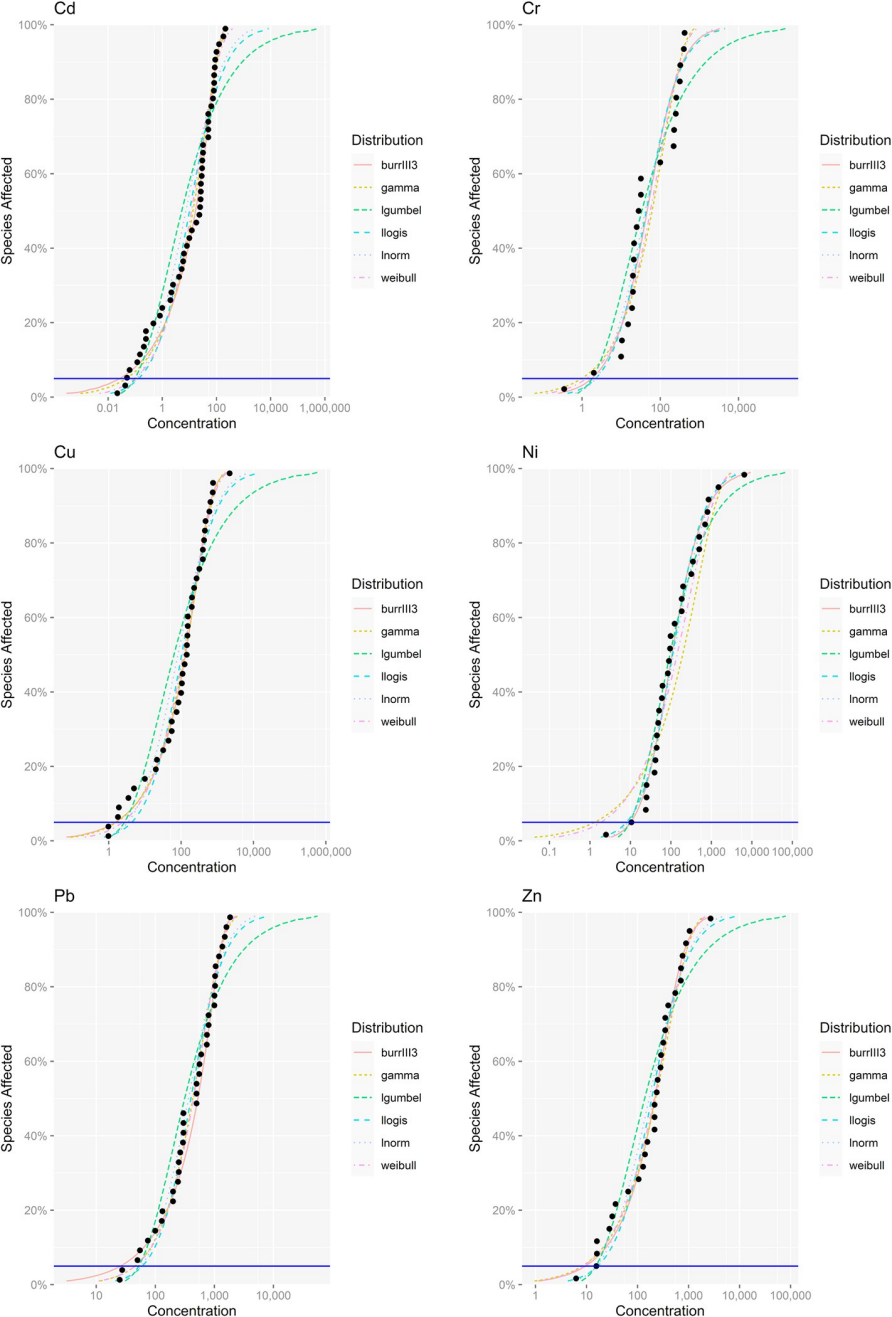
SSD curves of heavy metals(Cd, Cr, Ni, Cu, Pb and Zn) in soil organisms.

The fitting effects of heavy metals on SSD soil organisms are shown in Fig 6. Six types of models were fitted differently to the chronic toxicity data of heavy metals. The best one was chosen for use in this study.

HC_5_, as a significant threshold value, represents the maximum value of heavy metal concentration when 5% of the species are potentially threatened by toxicity (in other words, 95% of the species are protected). A smaller HC_5_ value means that soil organisms are more sensitive to changes in heavy metal concentrations in environmental media and are more likely to be endangered by toxicity. The order of HC_5_ values for each heavy metal in this study, from highest to lowest, was Pb, Zn, Ni, Cu, Cr and Cd, implying exactly the opposite order of ecological risk. Thus, Cd had the smallest HC_5_ value of 0.05 mg/kg at the NOEC/LOEC level, with the highest potential ecological risk. The HC_5_ value of Cd in this study was significantly lower than the recommended concentration limits of 0.15 mg/kg and 0.30 mg/kg proposed by some scholars for Cd in natural areas and agro-pastoral areas in China, respectively [44]. These gaps are probably the result of different protection targets and risk control levels.

HC_5_ has been commonly used in the past as an important basis for the development of environmental quality standards. The concentration limits of heavy metals in the soil environmental quality standards in force in several countries are listed in Table 4. There are differences in soil environmental quality standards among countries due to differences in protection targets, land use classification, exposure pathway settings, toxicological data sources, evaluation methods and soil types [45]. The rather obvious trend from the results of this study was that the limits of *risk control standard for soil contamination of agricultural land of China* (GB 15618–2018) aimed at ensuring the quality and safety of agricultural products, the normal growth of crops and the soil ecological environment and *risk control standard for soil contamination of development land of China* (GB 36600–2018) lain in controlling the risk of contaminated land to human health and to protect the safety of human living environment for heavy metals were generally higher than their ecological risk thresholds calculated by SSD method according to the technical guideline for deriving soil environmental criteria of China published by the Ministry of Ecology and Environment of China in this study, which may implied that soil organisms are potentially at risk of toxicity from the current state of heavy metal exposure, furthermore, the management standards of China concerned are poorly prevented for the ecological risk of heavy metals to soil organisms and need to be optimized.

**Table 4:** Limit values of trace elements in soil by country(mg/kg).

Heavy metal	Canada	Netherlands (target/intervention values)	Australia (health/ecological survey value)	United Kingdom	USA (eco-screening Value)	China (risk control standard for soil contamination of agricultural land)	China (risk control standard for soil contamination of development land)	HC_5_ from this study
**Cd**	1.4	0.6/1.2	20/3	1	32	0.3	65	0.05
**Cr**	64		100/1	130	-	200	5.7	1.3
**Cu**	63	40/190	1000/-	-	70	100	18000	1.64
**Ni**	-	-	600/60	50	38	100	900	8.04
**Pb**	70	50/530	300/600	-	120	120	800	45.69
**Zn**	200	140/720	7000/200	-	160	250	-	16.73

*- no data

#### Differential sensitivity of soil organisms to heavy metal toxicity

**Fig 7:**
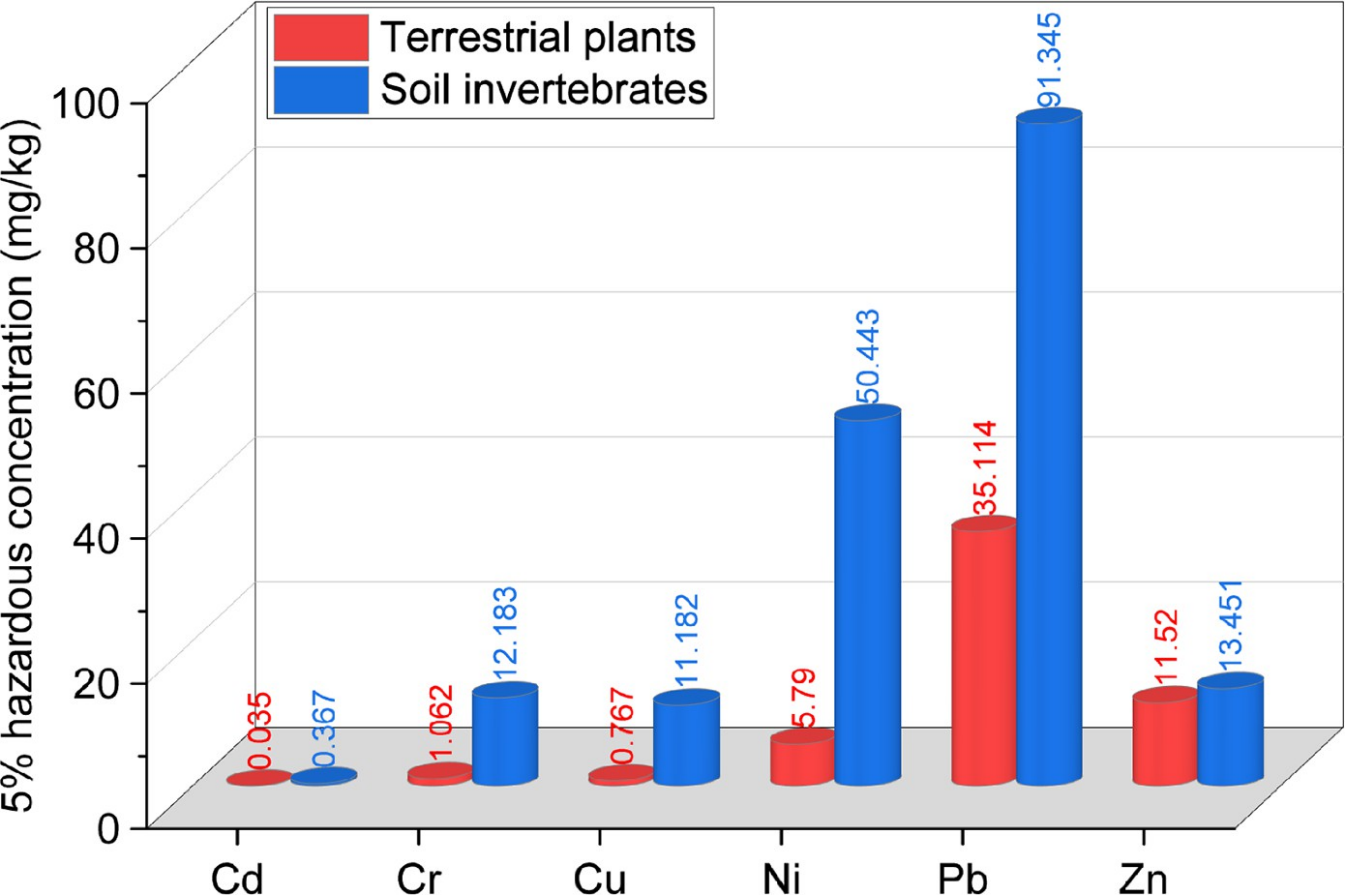
5% hazardous concentration for different soil organisms.

Fig 7 illustrates the HC_5_ of the NOEC/LOEC toxicological effects of heavy metals on terrestrial plants and soil invertebrates calculated with nonparametric methods. The general trend shows that the HC_5_ of heavy metals, including Cd, Cr, Cu, Ni, Pb and Zn, based on soil invertebrates were higher than those based on terrestrial plants, so it can be concluded that terrestrial plants are more sensitive to the potential toxicity of heavy metals and more susceptible to toxicity under conditions of lower heavy metal exposure concentrations. Depending on the HC_5_ values of the different heavy metals to terrestrial plants, the order from highest to lowest was Pb, Zn, Ni, Cr, Cu and Cd, while the HC_5_ based on soil invertebrates in decreasing order was Pb, Ni, Zn, Cr, Cu and Cd, from which it follows that both terrestrial plants and soil invertebrates are most sensitive to the potential toxic hazards of Cd. The excess enrichment of Cd in soils can cause a series of ecological and environmental issues. Cd is a highly bioaccumulative toxic metal element. Currently, data from soil, animal and plant surveys suggest potential Cd exposure risks. Once plants absorb excess Cd from soils, they will stress the germination of roots and seeds, inhibit the absorption of nutrition elements, alter the stomatal conductance of leaves, and affect ecosystem primary production [13].

**Fig 8:**
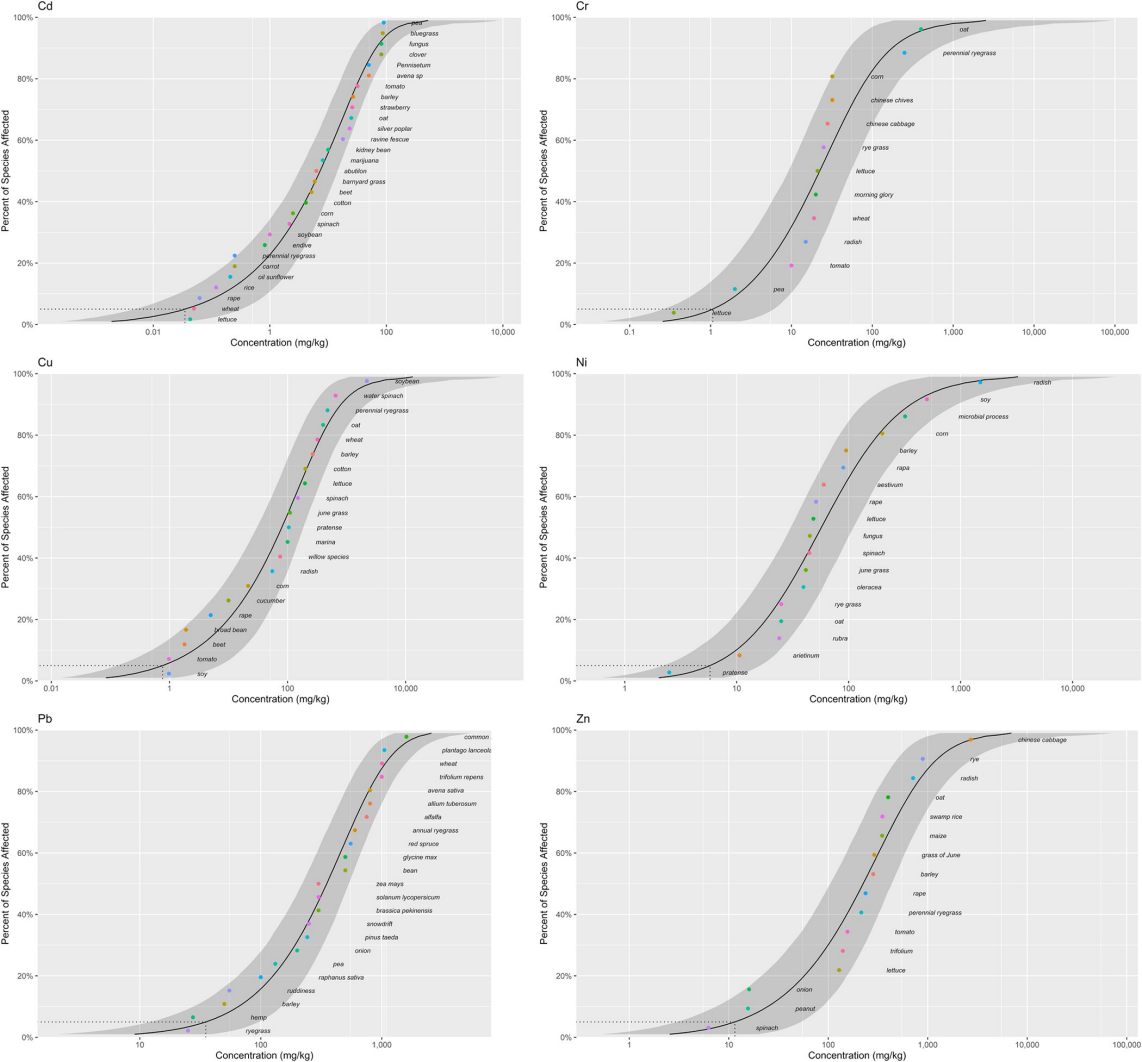
SSD curves of heavy metals(Cd, Cr, Ni, Cu, Pb and Zn) in terrestrial plants.

**Fig 9:**
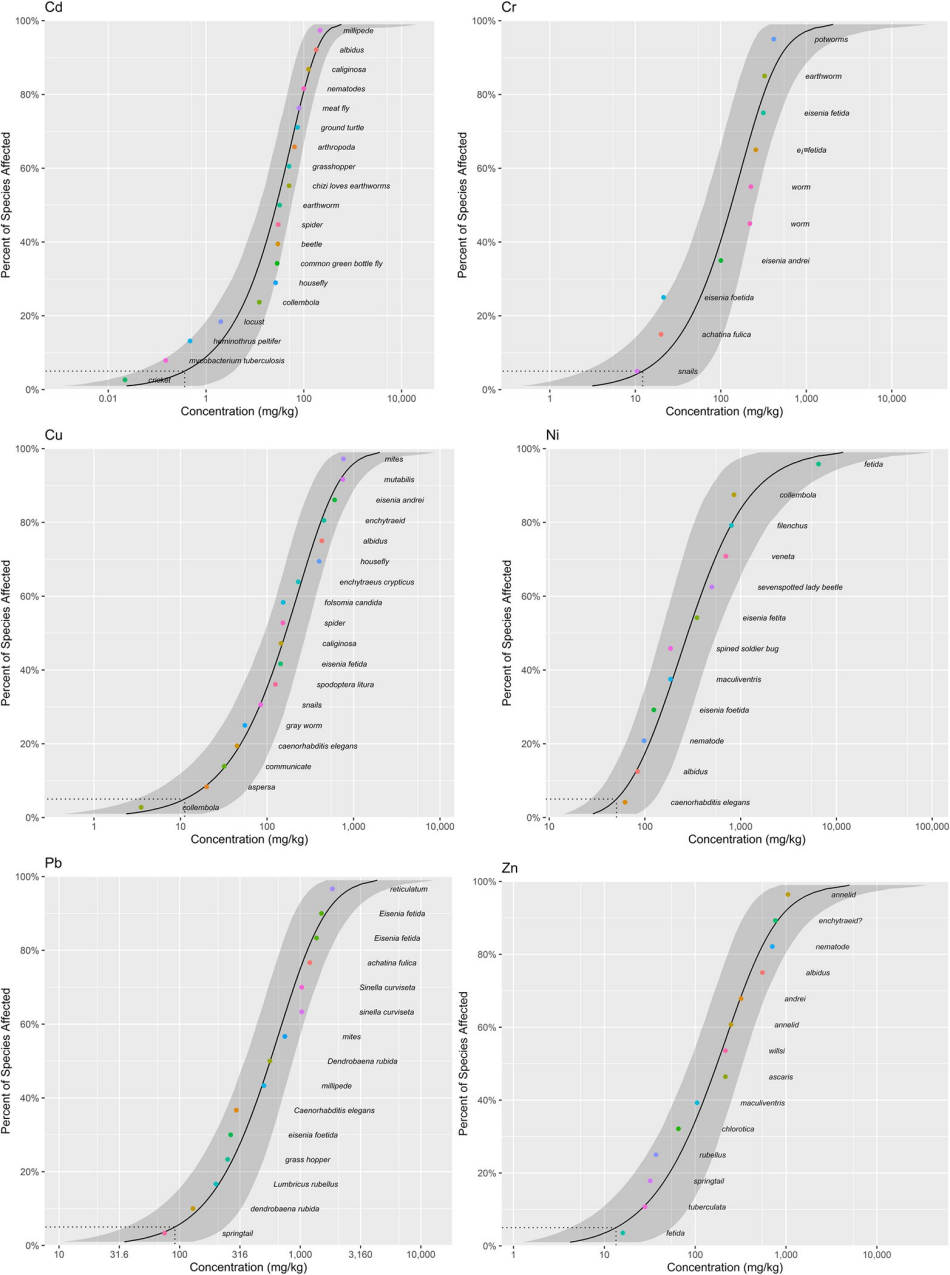
SSD curves of heavy metals (Cd, Cr, Ni, Cu, Pb and Zn) in soil invertebrates.

HC_5_ reflects the tolerance thresholds of soil organisms to heavy metals at low concentrations, while the SSD curves provide a visual representation of the differences in the sensitivity of species to heavy metals in varying concentration ranges [46]. As shown in Figs 8 and 9, the corresponding SSD curves of terrestrial plants and soil invertebrates showed basically the same trend in different heavy metal exposure concentration ranges. However, the ecological risks of different heavy metals for soil organisms still indicated local variations. The SSD curves for Cd, Cr, Cu, Ni and Pb were steeper for terrestrial plants at different exposure concentrations, most likely implying that the toxicity risks of Cd, Cr, Cu, Ni and Pb were higher for terrestrial plants than for soil invertebrates. For Zn, at relatively low exposure concentrations of no more than 100 mg/kg, it posed a greater ecological risk to terrestrial plants than to soil invertebrates. As the exposure concentration increased, the opposite trend was observed, with soil invertebrates posing a greater ecological risk than terrestrial plants.

#### Spatial variation in ecological risk

**Fig 10:**
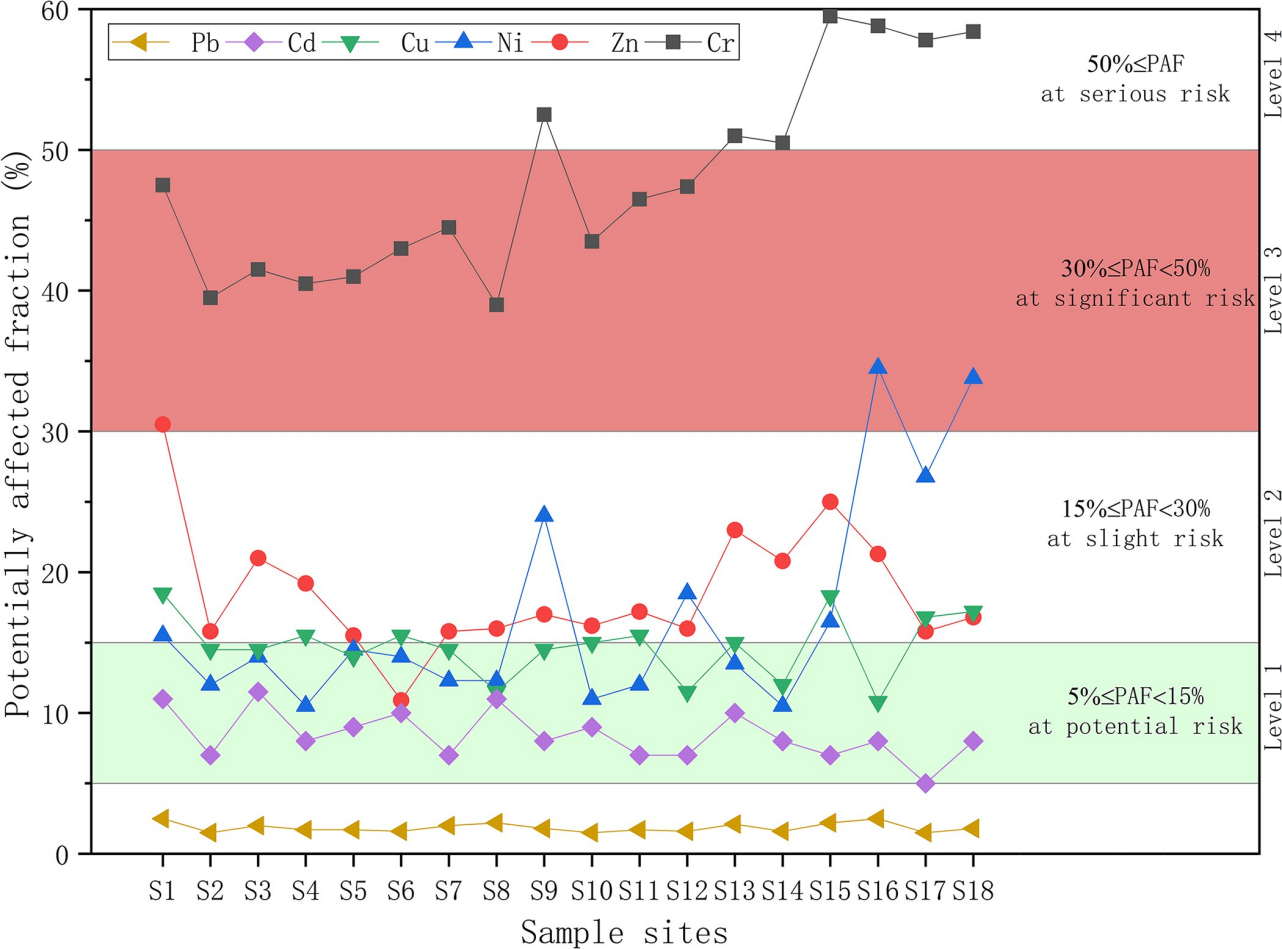
The potentially affected fraction of heavy metal exposure concentration.

PAF was calculated according to the SSD model with the best fitting effect, and the results are presented in Fig 10. In general, except for Cr, which had a greater ecological risk at all sample sites, Ni and Zn exhibited ecological risk level 3 at individual points, and the ecological risk of all the rest of the heavy metal exposure concentrations was low, with PAFs less than 30%, namely, they exhibited only potential or slight risk, especially Pb, which had PAF values all less than 5%. Several reasons can contribute to the PAFs of Cr, possibly because Cr is toxic to microorganisms and inhibits their metabolism, which reduces their number and further affects the microbial population abundance and community structure. Moreover, Cr inhibits the metabolism of soil animals, affects their reproduction, and leads to their death in serious cases. Finally, the lack of toxicological data of Cr affects the reliability of the SSD model. The PAFs of Ni in S18 reached 33.8% under long-term exposure conditions with significant risk, i.e., Level III. Sun showed that the accumulation of Ni in soil in China had an increasing trend, and the main sources were atmospheric deposition, livestock manure, fertilizer, sewage and sludge [47]. It was speculated that S18 was located in Renlongba Glacier with a maximum elevation of 5900 m, and the biotoxicity of Ni was affected due to the deposition of heavy metal pollutants emitted by human activities through atmospheric transportation.

## Conclusion

In recent years, in the context of global warming, with the increasing residential and tourist populations and rising traffic flow, superimposed on the flourishing of traditional industries such as agriculture and mining, the heavy metal pollution in the glacier area from Ranwu town to Renlongba on TP has become increasingly serious, and soil organisms are facing unprecedented potential ecological risks.

In terms of pollution status, the average levels of Cd, Ni and Sb were higher than the background levels, especially, Cd whose concentration exceeded the regional soil background value by more than four times, and the maximum EF value was as high as 7.69. Impressively, the sampling sites located near the hitherto undeveloped Renlungba Glacier showed relatively high contamination levels of Sb and Ni due to deposition after long-distance atmospheric transport, precipitation or release during glacial melting due to climate change.

From the 5% hazard concentration control level, HC_5_ values were lower for all terrestrial plants than for invertebrates. Among them, the HC_5_ value of Cd reached 0.05 mg/kg, showing the highest potential ecological risk. Terrestrial plants were more sensitive to Cd, Cr, Cu, Ni and Pb than invertebrates base on SSD curves trend. In terms of the cumulative probability of ecological risk based on PAF, Pb, Cd and Cu showed a relatively safe risk status, with PAF values in level I or II at all sites. The PAF values for Ni increased at sites close to the Renlungba glacier, reaching the highest value of 33.8%.

As for soil quality management strategy for heavy metal pollution, on the one side the development of environmental benchmarking for soil ecological safety based on the protection of soil organisms is urgently needed, especially for Cr, Zn and Ni ecological risk control standards; On the other side, comparing the HC_5_ values with the heavy metal concentration limits in the current Chinese soil environmental quality for agricultural and development land, the current management regime lacks sufficient control over the potential ecological risk of heavy metals to which soil organisms are exposed and need to be optimized.

## References

[1] Du HL, Wang Y, Wang JS, Yao YB, Zhou Y, Liu XY, et al. Distribution Characteristics and Ecological Risk Assessment of Soil Heavy Metals in Typical Watersheds of the Qinghai-Tibet Plateau. Huan Jing Ke Xue. 2021;42(9):4422–31. Epub 2021/08/21. doi: 10.13227/j.hjkx.202012123 34414742

[2] Tian M, Wang X, Liu F, Hu Q, Qiao Y, Wang Q. Spatial-temporal variability and influence factors of Cd in soils of Guangxi, China. PloS one. 2023;18(1):e0279980–e. doi: 10.1371/journal.pone.0279980 36626378 PMC9831335

[3] Chen NC, Zheng JY, He XF. Analysis of the bulletin of national soil pollution survey. Journal of Agro-Environment Science. 2017;36(9):1689–92. 10.1016/j.marpolbul.2013.05.026.

[4] He YQ. Distribution Characteristics and Pollution Assessment of Heavy Metals in Wounded Soils of Erlang Mountain Highway under Different Restoration Methods. Hans Journal of Soil Science. 2019;07(04):275–85. 10.1Z2677/hjss.2019.74034.

[5] Kuerban M, Maihemuti B, Waili Y, Tuerhong T. Ecological risk assessment and source identification of heavy metal pollution in vegetable bases of Urumqi, China, using the positive matrix factorization (PMF) method. Plos One. 2020;15(4). doi: 10.1371/journal.pone.0230191 32282796 PMC7153853

[6] Chen B, Tan SD, Dong FX, Yang YT. The toxicity of heavy metals to plants and the detoxification mechanism of plants to their toxicity. jiangsu agricultural sciences. 2019;47(04):34–8. 10.15889/j.issn.1002-1302.2019.04.007.

[7] Li X, Zheng LP, Zhang Y, Feng Y, Du J, Sun L, et al. Application of species sensitive distribution method to establish lead ecological safety soil environmental criteria. Journal of Ecotoxicology. 2021;16(01):107–18. 10.7524/AJE.1673-5897.20200630001.

[8] Sun YF, Wang GL, Liu CZ. Effects of heavy metal pollution on invertebrate community structure in farmland soil. Chinese Journal of Soil Science. 2014;45(01):210–5. 10.19336/j.cnki.trtb.2014.01.036.

[9] Zhang H, Zhang F, Song J, Tan ML, Kung H-t, Johnson VC. Pollutant source, ecological and human health risks assessment of heavy metals in soils from coal mining areas in Xinjiang, China. Environmental Research. 2021;202. 10.1016/j.envres.2021.111702. 34284019

[10] Kafle HK, Khadgi J, Ojha RB, Santoso M. Concentration, Sources, and Associated Risks of Trace Elements in the Surface Soil of Kathmandu Valley, Nepal. Water Air and Soil Pollution. 2022;233(2). 10.1007/s11270-021-05444-1.

[11] Wang X, Sun Y, Li S, Wang H. Spatial distribution and ecological risk assessment of heavy metals in soil from the Raoyanghe Wetland, China. Plos One. 2019;14(8). doi: 10.1371/journal.pone.0220409 31398209 PMC6688808

[12] Xiao Y, Guo M, Li X, Luo X, Pan R, Ouyang T. Spatial distribution, pollution, and health risk assessment of heavy metal in agricultural surface soil for the Guangzhou-Foshan urban zone, South China. Plos One. 2020;15(10). doi: 10.1371/journal.pone.0239563 33031419 PMC7544098

[13] Wang Z, Bing H, Zhu H, Wu Y. Fractions, Contamination and Health Risk of Cadmium in Alpine Soils on the Gongga Mountain, Eastern Tibetan Plateau. Bull Environ Contam Toxicol. 2021;106(1):86–91. Epub 2021/01/22. doi: 10.1007/s00128-020-03073-8 33475791

[14] Park TJ, Lee JH, Lee MS, Park CH, Lee CH, Moon SD, et al. Development of water quality criteria of ammonia for protecting aquatic life in freshwater using species sensitivity distribution method. Science of The Total Environment. 2018;634:934–40. doi: 10.1016/j.scitotenv.2018.04.018 29660887

[15] Shi BL, Zhang XH, Gou AP, editors. Research on heavy metal pollution remediation technology in farmland soil. 2nd International Conference on Geoscience and Environmental Chemistry (ICGEC); 2020;10(09). 10.1051/e3sconf/202020602011.

[16] Araujo PRM, Biondi CM, do Nascimento CWA, da Silva FBV, da Silva WR, da Silva FL, et al. Assessing the spatial distribution and ecologic and human health risks in mangrove soils polluted by Hg in northeastern Brazil. Chemosphere. 2021;266. doi: 10.1016/j.chemosphere.2020.129019 33272678

[17] Yang A, Wang YH, Hu J, Liu XL, Li J. Evaluation and Source of Heavy Metal Pollution in Surface Soil of Qinghai-Tibet Plateau. Huanjing Kexue. 2020;41(2):886–94. doi: 10.13227/j.hjkx.201907195 32608750

[18] Jiao XY, Dong ZW, Kang SC, Li YF, Jiang C, Rostami M. New insights into heavy metal elements deposition in the snowpacks of mountain glaciers in the eastern Tibetan Plateau. Ecotoxicology and Environmental Safety. 2021;207. doi: 10.1016/j.ecoenv.2020.111228 32890952

[19] Xiang L. Characteristics of Heavy Metal Content in Soil with Different Parent Materials—Taking Dongzhi County as an Example. anhui agricultural sciences. 2021;49(06):76–9. 10.3969/j.issn.0517-6611.2021.06.022.

[20] An S, Liu N, Li X, Zeng S, Wang X, Wang D. Understanding heavy metal accumulation in roadside soils along major roads in the Tibet Plateau. Sci Total Environ. 2021;802:149865. Epub 2021/08/30. doi: 10.1016/j.scitotenv.2021.149865 34455271

[21] Zhao ZY, Deng X, Han X, Xu ZH, Yu X. Characteristics and changes of Renlongba Glacier in Basu County, Tibet. technological innovation and application. 2020;(07):75–8. https://kns.cnki.net/KXReader/Detail?invoice=rj9p6ulh4E8IRfXGaWJszoG5S2yRzafJQQ6HpqUBs78dE9FkrBxLmyr2QhqzE%2FPMjai8Yi9U55RaTr%2BMmElENm82lXZIO77KHxi64ftMB4bkpPSc%2Fll8oovce2IYmak%2FvHUepweKR5j%2B%2FfwyUp5HLpe8FxG2%2BkDACDczkLXUJRs%3D&DBCODE=CJFD&FileName=CXYY202007028&TABLEName=cjfdlast2020&nonce=E0A6F42E0ABD4DD3AD35A1DB0E248806&uid=&TIMESTAMP=1678372352712#

[22] Lu DM, Peng MK. Comparison of different pretreatment methods for determination of lead, cadmium, chromium and arsenic in soil by ICP-MS. chemical industry management. 2021;(33):134–5. 10.19900/j.cnki.ISSN1008-4800.2021.33.063.

[23] Sun R, Chen L. Assessment of Heavy Metal Pollution in Topsoil around Beijing Metropolis. Plos One. 2016;11(5). doi: 10.1371/journal.pone.0155350 27159454 PMC4861295

[24] Tepe Y, Simsek A, Ustaoglu F, Tas B. Spatial-temporal distribution and pollution indices of heavy metals in the Turnasuyu Stream sediment, Turkey. Environmental Monitoring and Assessment. 2022;194(11). doi: 10.1007/s10661-022-10490-1 36136175

[25] Ustaoglu F, Tepe Y, Aydin H. Heavy metals in sediments of two nearby streams from Southeastern Black Sea coast: Contamination and ecological risk assessment. Environmental Forensics. 2020;21(2):145–56. 10.1080/15275922.2020.1728433.

[26] Luo Q, Zhang ZM, Xiang Z, He HZ. Distribution and Enrichment Characteristics of Heavy Metals in Soil of Woodland in Fanjing Mountain Nature Reserve. Southwest China Journal of Agricultural Sciences. 2017;30(10):2352–9. 10.16213/j.cnki.scjas.2017.10.032.

[27] Thorley J, Dalgarno S, Schwarz C. SSDCA: An R package and web page to calculate species sensitivity distributions (PL). Canadian Technical Report of Fisheries and Aquatic Sciences. 2020;3360:79–. BCI:BCI202000609151.

[28] Burnham KP, Anderson DR. Multimodel inference—understanding AIC and BIC in model selection. Sociological Methods & Research. 2004;33(2):261–304. 10.1177/0049124104268644.

[29] Yan ZG, Liu ZT, Meng W. Development of emergency water quality standards for Cr6+and Hg2+in Liaohe River basin. Engineering Science of China. 2013;15(03):26–32. 10.3969/j.issn.1009-1742.2013.03.006.

[30] Bing H. Mobility and eco-risk of trace metals in soils at the Hailuogou Glacier foreland in eastern Tibetan Plateau. Environ Sci Pollut Res Int. 2016;23(6):5721–32. Epub 2015/11/20. doi: 10.1007/s11356-015-5592-2 26581692

[31] Hou L, Li WB. Potential ecological risk of heavy metals in soil beside new roads in alpine ecologically fragile areas. Journal of Safety and Environment. 2021;21(04):1832–1838. 10.13637/j.issn.1009-6094.2020.0623.

[32] Chen W, Li Q, Wang Z, Sun Z. Spatial distribution characteristics and pollution assessment of heavy metals in farmland soils in China. Environmental Science & Technology. 2020;41(06):2822–33. 10.13227/j.hjkx.201910075. 32608799

[33] Grzebisz W, Ciesla L, Komisarek J, Potarzycki J. Geochemical assessment of the heavy metals pollution of urban soils. Polish Journal of Environmental Studies. 2002;11(5):493–500. 10.1007/BF03326197.

[34] Silva HF, Silva NF, Oliveira CM, Matos MJ. Heavy Metals Contamination of Urban Soils-A Decade Study in the City of Lisbon, Portugal. Soil Systems. 2021;5(2). 10.3390/soilsystems5020027.

[35] Dœlsch E, Van de Kerchove V, Saint Macary H. Heavy metal content in soils of Réunion (Indian Ocean). Geoderma. 2006;134(1–2):119–34. 10.1016/j.geoderma.2005.09.003.

[36] Kim S-R, Song S-T, Lee M-G, Kam S-K, 현성수. Concentration of Heavy Metals in Natural Soils of Jeju Island, Korea. Journal of Environmental Science International. 2015;24(2):175–88. 10.5322/jesi.2015.24.2.175.

[37] Chandrasekaran A, Ravisankar R, Harikrishnan N, Satapathy KK, Prasad MVR, Kanagasabapathy KV. Multivariate statistical analysis of heavy metal concentration in soils of Yelagiri Hills, Tamilnadu, India—Spectroscopical approach. Spectrochimica Acta Part a-Molecular and Biomolecular Spectroscopy. 2015;137:589–600. doi: 10.1016/j.saa.2014.08.093 25240831

[38] Wilcke W, Muller S, Kanchanakool N, Zech W. Urban soil contamination in Bangkok: heavy metal and aluminium partitioning in topsoils. Geoderma. 1998;86(3–4):211–28. 10.1016/s0016-7061(98)00045-7.

[39] Wang YH. Distribution characteristics, sources and ecological risk assessment of heavy metals in soil of Qinghai-Tibet Plateau [Master]: tianjin normal university; 2018. https://d.wanfangdata.com.cn/thesis/ChJUaGVzaXNOZXdTMjAyMzAxMTASCFkzNDk4OTUyGghmYWw2d3cyZQ%3D%3D

[40] Yang A, Wang YH, Hu J, Liu XL, Li J. Evaluation and source analysis of heavy metal pollution in topsoil of Qinghai-Tibet Plateau. environmental Science & Technology. 2020;41(02):886–94. 10.13227/j.hjkx.201907195. 32608750

[41] Lin J, Qiu QR, Chen JA, Zhang ZC, Du ZX, Zhang Q. Evaluation of heavy metal and metalloid pollution in roadside soil. Journal of Environment andHealth. 2000;(05):284–6. 10.16241/j.cnki.1001-5914.2000.05.015.

[42] Xue HH, Chen WL, Li M, Liu BS, Li G, Han XK. Assessment of major ions and trace elements in snow: A case study across northeastern China, 2017–2018. Chemosphere. 2020;251. 10.1016/j.chemosphere.2020.126328. 32169706

[43] McConnell JR, Edwards R. Coal burning leaves toxic heavy metal legacy in the Arctic. Proceedings of the National Academy of Sciences of the United States of America. 2008;105(34):12140–4. doi: 10.1073/pnas.0803564105 18711138 PMC2529165

[44] Wu YY, Zhou QX, Adriano DC. Interim environmental guidelines for cadmium and mercury in soils of China. Water, Air, and Soil Pollution. 1991;(57–1). 10.1007/BF00282937.10.1007/BF00282937.

[45] Zhang H, Luo Y, Xia J, Zhang H. International comparison and enlightenment of risk-based soil environmental quality standards. environmental Science & Technology. 2011;32(03):795–802. 10.13227/j.hjkx.2011.03.025.

[46] Kong XZ, He W, Qin L, He FS, Wang Y. Assessment of species sensitivity distribution of heavy metals to freshwater biological ecological risk. china environmental science. 2011;31(09):647–54. https://doi.org/CNKI:SUN:ZGHJ.0.2011-09-033.

[47] Sun C, Chen S, Ma Y, Liu J. Ecological Hazard Concentration(HC_5)of Cadmium(Cd)to Rice Cultivars Under Hydroponic Culture as Determined with Species Sensitivity Distribution Model(Burr-Ⅲ). Journal of Agro-Environment Science. 2013;32(12):2316–22. 10.11654/jaes.2013.12.002.

